# Digital Health Literacy, Perceived Benefits, and Health-Promoting Behaviors Among Transition-Age Young Adults with Developmental Disabilities

**DOI:** 10.3390/healthcare14213579

**Published:** 2026-10-22

**Authors:** Kihee Kim, Suyon Baek

**Affiliations:** 1 Boryeong Jeongsim Special School, Boryeong-si 33447, Republic of Korea; b99022@jeongsim.cnesc.kr; 2 Department of Nursing, Graduate School, Kongju National University, Gongju-si 32588, Republic of Korea; 3 Department of Nursing, College of Nursing and Health, Kongju National University, Gongju-si 32588, Republic of Korea; 4 Nursing Convergence Research Center, Kongju National University, Gongju-si 32588, Republic of Korea

**Keywords:** developmental disabilities, transition-age young adults, digital health literacy, perceived benefits, health-promoting behaviors

## Abstract

**Highlights:**

**What are the main findings?**

- Perceived benefits and digital health literacy were significantly associated with health-promoting behaviors among transition-age young adults with developmental disabilities.
- Digital health literacy scores showed modest variation across subdomains, with searching having the highest mean and self-expression having the lowest mean, while perceived benefits were rated highly.

**What are the implications of the main findings?**

- Programs may consider addressing digital health literacy, particularly evaluation and self-expression skills, to support effective engagement in health-promoting behaviors.
- Future studies should examine whether structured environmental support strengthens the role of perceived benefits in health management participation.

**Abstract:**

Background/Objectives: This study examined the associations of digital health literacy and perceived benefits with health-promoting behaviors among transition-age young adults with developmental disabilities. Methods: A cross-sectional survey was conducted with 137 participants recruited from six special schools and six social welfare facilities in C province (8 July–30 September 2025). Participants completed self-report measures of digital health literacy, perceived benefits, and health-promoting behaviors. Data were analyzed using Pearson’s correlations and hierarchical multiple regression, controlling for gender, age, type of disability, and independent digital health information-searching ability. Results: Mean scores were 2.47 for digital health literacy (1–4), 4.03 for perceived benefits (1–5), and 3.91 for health-promoting behaviors (1–5). Health-promoting behaviors correlated positively with digital health literacy (*r* = 0.50, *p* < 0.001) and perceived benefits (*r* = 0.68, *p* < 0.001). The Step 1 model, which contained only the control variables, was not significant (R^2^ = 0.05). Adding digital health literacy and perceived benefits significantly improved the model (Adj. R^2^ = 0.52; ΔR^2^ = 0.50). Digital health literacy (β = 0.36, *p* < 0.001) and perceived benefits (β = 0.56, *p* < 0.001) were significantly associated with health-promoting behaviors. Conclusions: Digital competencies and motivational cognitions, particularly perceived benefits, may be important factors associated with health-promoting behaviors in transition-age young adults with developmental disabilities. Health promotion programs may consider reinforcing perceived benefits through repeated, concrete experiences and feedback, and enhancing digital health literacy beyond basic searching through supported training in evaluation and self-expression skills. Future intervention studies should examine whether structured support environments enhance the effects of perceived benefits and digital health literacy training on health-promoting behaviors.

## 1. Introduction

Developmental disability refers to a condition requiring support in daily life due to limitations in developmental areas such as cognition, social skills, communication, motor skills, and adaptive functioning. In Korean law and service provision, developmental disability comprises intellectual disability and autism spectrum disorder. This grouping relates to the broader international concept of “Intellectual and Developmental Disabilities (IDD)” [1,2]. Individuals with developmental disabilities often require ongoing support across various fields, including education, social welfare, healthcare, and employment [3,4,5].

Transition to adulthood (ages 19–24) is a critical developmental period for identity formation, relationship development, career choice, and increasing economic and emotional independence [6]. For young adults with developmental disabilities, successful transition during this period is particularly important because it is closely linked to adult outcomes such as employment, independent living, and quality of life [7].

According to the 2021 Survey on Persons with Developmental Disabilities in Korea, chronic disease prevalence is higher among individuals with developmental disabilities (54.4%) compared to the general adult population (38.9%). However, their participation in health screenings (50.2%) and regular exercise (25.7%) is substantially lower than that of the general adult population (74.2% and 48.8%, respectively), indicating overall lower engagement in health-promoting behaviors [8,9,10]. International evidence similarly shows higher rates of chronic and mental health conditions and lower engagement in health-promoting behaviors among individuals with developmental disabilities, and recent studies suggest these disparities persist [11,12]. Transition-age young adults with developmental disabilities are also considered a high-risk group due to service gaps and disrupted continuity of care during the transition to adulthood, particularly when moving from pediatric to adult healthcare and social welfare systems [13].

Health-promoting behaviors are actions taken to maintain and enhance health, such as physical activity, nutrition management, and stress regulation [14]. However, among individuals with developmental disabilities, research on health-promoting behaviors remains limited and fragmented. Previous studies have mainly focused on selected risk behaviors (e.g., smoking, alcohol use, overweight/obesity, and stress) [15] or physical activity participation [16,17] rather than overall health-promoting behaviors. In addition, few studies have specifically addressed the transition-to-adulthood period [15,16] and analyses primarily concern life-stage or gender differences [15]. Additionally, many studies have relied on caregiver proxy reports rather than self-reports [16,17], potentially underrepresenting individuals’ own experiences and relevant determinants.

Since the COVID-19 pandemic, rapid digitalization has shifted health information and services toward digital platforms, including telemedicine, kiosk-based registration, and mobile app-based health management. Accordingly, digital health literacy, defined as the ability to search for, understand, evaluate, and apply health information through digital technologies, has become essential for engaging in health behaviors [18,19]. In the present study, this broad competency was examined while acknowledging that, for transition-age young adults, digital health information access and communication are often practiced through mobile health contexts, including smartphones, mobile applications, and mobile-based communication platforms [20,21]. Individuals with developmental disabilities reportedly have substantially lower digital health literacy compared to those with physical disabilities and those without disabilities [22]. Recent international studies among this population further indicate persistent difficulties in understanding online health information [23] and the limited use of available online resources [24,25]. This digital health literacy gap may reflect contextual factors, such as socioeconomic conditions and the digital skills of family members or caregivers, and may in turn restrict access to health information and exacerbate health inequalities [26,27].

Perceived benefits refer to beliefs about the positive aspects or expected outcomes of engaging in health-promoting behaviors [28]. In this study, perceived benefits represent the extent to which transition-age young adults with developmental disabilities believe that maintaining a healthy lifestyle is beneficial to their health and daily lives. These beliefs are important because they may motivate engagement in health-promoting behaviors. This concept is particularly relevant in the present study, as transition-age young adults with developmental disabilities may have difficulty anticipating long-term benefits due to cognitive and communication limitations [27,29]. Therefore, interventions that enhance perceived benefits through repeated experiences of concrete gains and systematic presentation of these benefits may be critical for promoting health behaviors in this population.

Health disparities among people with intellectual and developmental disabilities are well documented and include a higher chronic disease burden, lower engagement in preventive care, and barriers to health-related self-management [9,10,11,12,27]. Within this literature, studies on health promotion have often focused on individual health behaviors—physical activity, weight management, smoking, or selected risk behaviors—rather than on overall health-promoting behaviors [15,16,17]. Another line of research treats the transition to adulthood as a period in which young people with developmental disabilities may experience reduced structure and discontinuity in education-, welfare-, and healthcare-based support [6,13]. Meanwhile, digital health literacy has grown in importance as health information and services are increasingly delivered through online, mobile, and platform-based environments [20,21,23]. However, recent reviews indicate that this research remains limited for people with intellectual disabilities and has concentrated on functional access and understanding, with less attention to higher-order skills such as appraising, applying, and communicating health information [23,25]. In parallel, health promotion research identifies perceived benefits as an important motivational belief underlying engagement in health behaviors [14,28]. Despite these developments, few studies have examined digital health literacy and perceived benefits together in relation to overall health-promoting behaviors among transition-age young adults with developmental disabilities [15,16,17,22,23].

Therefore, this study aimed to assess digital health literacy, perceived benefits, and health-promoting behaviors among transition-age young adults with developmental disabilities and to examine the associations among these variables in the developmental and support context of this population. By focusing on a transition-age cohort and using self-reported data, this study provides evidence from the perspectives of young adults with developmental disabilities themselves and may inform the development of context-sensitive strategies to support health-promoting behaviors in digital environments. Specifically, this study aimed to identify the following:

1. Participants’ general characteristics.
2. Levels of digital health literacy, perceived benefits, and health-promoting behaviors among the participants.
3. Correlations among digital health literacy, perceived benefits, and health-promoting behaviors.
4. Associations between digital health literacy, perceived benefits, and health-promoting behaviors.

## 2. Methods

### 2.1. Study Design

This study employed a descriptive correlational survey design to examine the associations of digital health literacy and perceived benefits with health-promoting behaviors among transition-age young adults with developmental disabilities.

### 2.2. Participants

Participants were transition-age young adults with developmental disabilities, defined as individuals aged 19–24 years living in the Republic of Korea [30]. Eligible individuals were those who could understand the study purpose and questionnaire items and independently complete the self-report questionnaire. This criterion was applied because the study aimed to collect data based on participants’ own perceptions and experiences. Both participants and legal guardians provided informed consent. The minimum sample size, calculated using G*Power 3.1.9 (Heinrich Heine University Düsseldorf, Düsseldorf, Germany) for multiple regression (power = 0.80, α = 0.05, effect size = 0.15, eight predictors), was 109 [31]. To account for 20% attrition, 140 questionnaires were distributed; after excluding three incomplete responses, 137 were analyzed.

### 2.3. Instruments

This study used a structured self-report questionnaire comprising digital health literacy (12 items), perceived benefits (8 items), health-promoting behaviors (26 items), and general characteristics (6 items). Permission to use each instrument was obtained from the original authors via email. The plain-language versions were used only to support comprehension and did not alter the original construct, item structure, or scoring. Seven experts (four special education teachers and three school health teachers) assessed content validity using a 4-point relevance scale and written feedback, focusing on the clarity and appropriateness of the plain-language wording and the suitability of applying instruments originally developed for school-age children to transition-age young adults with developmental disabilities. All 46 items met the content validity criteria (I-CVI = 1.00; S-CVI = 1.00).

#### 2.3.1. Digital Health Literacy

Digital health literacy was measured using the Digital Health Literacy Instrument (DHLI) developed by van der Vaart and Drossaert [18], validated in Korean college students by Chun et al. [32], and revised by Choi et al. [33]. The DHLI includes 12 items across four subdomains (searching, self-expression, evaluation, and application—three items each). An example item is “I can judge whether the health information I find online is reliable,” and its plain-language version is “I can tell whether the health information I find on the internet or a smartphone can be trusted.” Items are rated on a 4-point Likert scale (1 = “very difficult” to 4 = “very easy”), with higher scores indicating greater digital health literacy. Cronbach’s α for the full scale was 0.91 in Chun et al. (subdomains α = 0.77–0.86) [32] and α = 0.96 in the present study (subdomains α = 0.86–0.89).

#### 2.3.2. Perceived Benefits

Perceived benefits were measured using Cho and Kwon’s [34] revised version of the scale originally developed by Kim and Lee [35] for late school-age children. The instrument consists of 8 items rated on a 5-point Likert scale (1 = “not at all true” to 5 = “very true”), with higher scores indicating greater perceived benefits of engaging in health behaviors. An example item is “I think maintaining healthy lifestyle habits will help prevent disease,” and its plain-language version is “I think exercising and eating well will help me avoid getting sick.” Cronbach’s α was 0.71 at the time of development [35] and α = 0.77 in Cho and Kwon [34]; Cronbach’s α was 0.91 in the present study.

#### 2.3.3. Health-Promoting Behaviors

Health-promoting behaviors were measured using a scale developed by Kim and Lee [35] and revised by Park [36]. To improve comprehension for transition-age young adults with developmental disabilities, the school-age version adapted by Park [36] was used, emphasizing concrete daily behaviors. The 26-item instrument includes four subdomains: personal hygiene and lifestyle habits (9 items), accident prevention (6 items), infectious disease prevention (6 items), and mental health (5 items). An example item is “I undergo regular dental check-ups,” and its plain-language version is “I go to the dentist for a check-up at regular times even if my teeth do not hurt.” Items are rated on a 5-point Likert scale (1 = “not at all true” to 5 = “very true”), with higher scores indicating greater engagement in health-promoting behaviors. Cronbach’s α was 0.80 in Kim and Lee [35] and 0.87 in Park (subdomains α = 0.76–0.86) [36]; in the present study, Cronbach’s α was 0.92 (subdomains α = 0.71–0.83).

### 2.4. Data Collection

Data were collected between 8 July and 30 September 2025. Participants were recruited through convenience sampling from six special schools and six social welfare facilities (developmental disabilities support centers and sheltered workshops) in C province. After obtaining institutional approval via email, posters and notices were displayed in classrooms and common areas. In special schools, legal guardians received home packets containing information sheets and consent forms; in welfare facilities, internal notices were posted alongside the same materials for interested users. To standardize data collection, a teacher or staff member at each site served as a research assistant and received pre-training on eligibility criteria, questionnaire administration (including the plain-language version), allowable explanations, avoidance of leading responses, assistance procedures, and maintaining confidentiality. Disability type was determined from the primary registered disability type reported by participants or legal guardians, and where available, confirmed through institutional records, consistent with Korea’s disability registration system [37]. Where teachers or facility staff assisted, they verified the registered disability type held in institutional documents and made no independent diagnostic judgments. The researchers conducted no clinical diagnostic assessments. Participants registered with intellectual disability were assigned to the intellectual disability group, whereas those registered with autistic disorder or autism spectrum disorder were assigned to the autism spectrum disorder group. Participants were not asked to report multiple diagnoses or detailed co-occurring conditions. Accordingly, although intellectual disability and autism spectrum disorder are distinct diagnostic categories and may co-occur [38], the groups in this study should be interpreted as administrative, service-related categories based on primary registered disability type rather than as mutually exclusive clinical phenotypes. Surveys were administered individually in a quiet space and were primarily self-completed using either the original or plain-language version. Completion time averaged approximately 20–30 min. Although limited procedural assistance—as approved by the Institutional Review Board (e.g., reading items aloud or recording a participant’s stated response)—was permitted, no such assistance was required in the present study.

### 2.5. Data Analysis

The collected data were analyzed using IBM SPSS Statistics 27.0 (IBM Corp., Armonk, NY, USA) as follows.

1. Participants’ general characteristics and their levels of digital health literacy, perceived benefits, and health-promoting behaviors were analyzed using frequency, percentage, mean, and standard deviation (SD). For descriptive analyses, ages 19 and 20 were combined into a single category because few participants were aged 19. This grouping applies to Table 1 only; in the regression analysis, age was recoded as actual age in years, with values ranging from 19 to 24 years, and it was entered as a continuous variable.
2. Differences in health-promoting behaviors according to participants’ general characteristics were analyzed using independent t-tests and one-way analysis of variance (ANOVA), with Scheffé tests conducted for post hoc comparisons.
3. Correlations among digital health literacy, perceived benefits, and health-promoting behaviors were examined using Pearson’s correlation coefficients.
4. Factors associated with health-promoting behaviors were identified using hierarchical multiple regression analysis.

### 2.6. Ethical Considerations

This study was approved by the Institutional Review Board of Kongju National University (Approval No.: KNU_IRB_2025_051). Given that the participants were transition-age young adults with developmental disabilities, additional safeguards were implemented to ensure voluntary participation, respect for autonomy, and confidentiality. Participants and their legal guardians received separate written information sheets explaining the study purpose, procedures, expected time required, potential benefits and minimal risks, confidentiality protection, voluntary participation, and the right to withdraw at any time without penalty or disadvantage in education, services, or facility use. Written informed consent was obtained from both participants and their legal guardians. Participants were included only if they could understand the study purpose and questionnaire items and voluntarily express their willingness to participate. Research assistants provided explanations in plain language when needed, allowed sufficient time for decision-making, and ensured that participants could refuse to answer any item or discontinue the survey at any time. Teachers, facility staff, and legal guardians were instructed not to pressure participants or influence their responses. To protect confidentiality, questionnaires did not include personally identifiable information, and all data were coded and analyzed anonymously. Completed questionnaires were stored securely and used only for research purposes. Participants received a small gift after participation; its value was kept modest to minimize the possibility of undue influence.

## 3. Results

### 3.1. Participants’ General Characteristics and Corresponding Health-Promoting Behaviors

Of the participants, 86 (62.8%) were male, and the largest age group was 19–20 years (*n* = 45, 32.8%). Most had intellectual disabilities (*n* = 126, 92.0%). For weekly digital device use, “almost daily” was most common (*n* = 65, 47.5%), followed by “several times a day” (*n* = 31, 22.6%). Regarding digital health information seeking, “never” was the most frequent response (*n* = 54, 39.4%), followed by “about once a month” (*n* = 46, 33.6%). Nearly half (*n* = 68, 49.6%) reported being able to search for health information independently, whereas 37 (27.0%) required assistance and 32 (23.4%) were unable to do so independently. Health-promoting behaviors did not differ significantly by general characteristics (Table 1).

### 3.2. Levels of Digital Health Literacy, Perceived Benefits, and Health-Promoting Behaviors

The mean score for digital health literacy was 2.47 ± 0.78, the mean perceived benefits score was 4.03 ± 0.81, and the mean health-promoting behaviors score was 3.91 ± 0.66. For the subdomains of digital health literacy, the mean scores were 2.62 ± 0.86 for searching, 2.38 ± 0.84 for self-expression, 2.41 ± 0.84 for evaluation, and 2.47 ± 0.85 for application. For the subdomains of health-promoting behaviors, the mean score was 3.75 ± 0.75 for personal hygiene and lifestyle habits, 3.86 ± 0.79 for accident prevention, 4.15 ± 0.75 for infectious disease prevention and management, and 3.98 ± 0.85 for mental health (Table 2).

### 3.3. Correlations Among Digital Health Literacy, Perceived Benefits, and Health-Promoting Behaviors

Health-promoting behaviors were significantly and positively correlated with digital health literacy (*r* = 0.50, *p* < 0.001) and perceived benefits (*r* = 0.68, *p* < 0.001) (Table 3).

### 3.4. Factors Associated with Health-Promoting Behaviors

Before hierarchical regression, assumptions were examined. The Durbin–Watson statistic was 2.00, indicating no residual autocorrelation, and variance inflation factor values ranged from 1.05 to 1.79, indicating no multicollinearity. Visual inspection of the histogram and normal P–P plot of standardized residuals indicated that the residuals were approximately normally distributed. The scatterplot of standardized residuals against standardized predicted values showed no clear systematic pattern, suggesting that the homoscedasticity assumption was reasonably met. In Step 1, control variables were entered, with categorical variables dummy-coded, to estimate the associations of digital health literacy and perceived benefits more accurately. The control variables were gender, age, type of disability, recruitment setting type, and the ability to independently search for health information using digital devices, selected based on previous literature and their conceptual relevance to health-promoting behaviors [13,20,39,40]. Recruitment setting type was included to partially account for differences between special schools and social welfare facilities. Individual site identifiers were not retained in the analytic dataset; therefore, cluster-robust standard errors could not be estimated. In Step 2, digital health literacy and perceived benefits were added to the model.

The Step 1 model was not significant (F = 1.14, *p* = 0.341; R^2^ = 0.05, Adj. R^2^ = 0.01). After adding digital health literacy and perceived benefits, the model became statistically significant (F = 19.70, *p* < 0.001) and accounted for 55% of the variance (Adj. R^2^ = 0.52; ΔR^2^ = 0.50). In the final model, digital health literacy (β = 0.37, *p* < 0.001) and perceived benefits (β = 0.56, *p* < 0.001) were significantly associated with health-promoting behaviors, whereas control variables were not significant (Table 4).

## 4. Discussion

This study examined the associations of digital health literacy and perceived benefits with health-promoting behaviors among 137 transition-age young adults with developmental disabilities, aiming to provide foundational evidence for strategies to improve these behaviors.

The mean score of health-promoting behaviors in the present study was 3.91 ± 0.66 out of 5. Among the subdomains, infectious disease prevention and management scored highest (4.15), whereas personal hygiene and lifestyle scored lowest (3.75), suggesting a moderate-to-high overall level. Few prior studies have directly examined health-promoting behaviors in transition-age or young adults with developmental disabilities. A closely related study by Choi and Lee [7] reported a significant increase in health-promoting behavior scores among young adults with developmental disabilities, from 27.00 before the intervention to 30.44 out of 45 after the intervention. However, direct numerical comparison with that study should be made cautiously, because Choi and Lee used a parent-rated 15-item measure in a single-group pre-post intervention design, whereas the present study used a self-report 26-item measure in a cross-sectional design.

Lee et al. [41] noted that many individuals with developmental disabilities live in structured environments and receive repeated guidance from teachers or caregivers. Participants in this study were enrolled in special schools and social welfare facilities and received regular education and services; systematic support and training may therefore have contributed to these behaviors. At the same time, the study did not collect detailed information on participants’ exposure to health education, independent living programs, transition preparation, digital skills training, or other structured supports. The findings should therefore not be interpreted as purely individual-level outcomes. Rather, they may reflect both individual-level factors and the broader educational and service contexts in which participants were supported.

The transition-to-adulthood context further supports this interpretation. As young adults with developmental disabilities move from school-based environments into adult life, family support, educational experiences, transition services, and the continuity of welfare- and healthcare-based provision may all change [5,13]. These changes may create both opportunities and barriers for health-promoting behaviors. For example, greater autonomy may allow more practice in health self-management, whereas reduced structure, supervision, and routine guidance may make daily health behaviors harder to sustain. These environmental changes may arrive before the self-regulation skills needed to plan, implement, and monitor health behaviors are sufficiently developed. Therefore, maintaining health-promoting behaviors during this transition may require not only individual motivation and self-management skills but also continued family, educational, service, and healthcare support, together with structured opportunities to practice those skills [42]. This interpretation is consistent with a settings-based approach to health promotion, which emphasizes that healthy living among people with intellectual disabilities is shaped by social networks, accessible places, person–environment fit, healthcare and prevention resources, financial conditions, and organizational policies [43].

In addition, the relatively high score should be considered in relation to the measurement focus of the instrument. The health-promoting behavior scale used in this study emphasized concrete and routine behaviors, including personal hygiene, lifestyle habits, accident prevention, infectious disease prevention and management, and mental health. Therefore, the score may reflect foundational health routines that are repeatedly taught and supported in daily contexts, rather than comprehensive or fully independent health promotion across broader domains such as nutrition, sleep, physical activity, self-management, and health or service utilization [35,36,44,45]. Although the instrument demonstrated content validity through expert review, future research should develop and validate measures that more comprehensively capture health-promoting behaviors relevant to transition-age young adults with developmental disabilities.

In this study, the mean digital health literacy score among transition-age young adults with developmental disabilities was 2.47 out of 4. To our knowledge, no peer-reviewed study has specifically targeted transition-age young adults with developmental disabilities and quantitatively measured digital health literacy in a way directly comparable to the present study. Nevertheless, the present findings are broadly consistent with the closest available evidence. In South Korea, Bang et al. [22] reported that people with developmental disabilities had significantly lower general and COVID-related eHealth scores than people with physical disabilities and non-disabled individuals, suggesting a substantial digital health literacy disadvantage in this population.

By subdomain, “searching” scored highest, followed by “application,” “evaluation,” and “self-expression.” This suggests that digital health literacy among transition-age young adults with developmental disabilities may be concentrated in basic information-seeking, while higher-order competencies—critically evaluating information and communicating health status or needs digitally—are relatively weak. This pattern is consistent with international digital health literacy frameworks that conceptualize digital health literacy as extending beyond basic information searching to include higher-order skills such as evaluating information, determining relevance, and communicating in digital environments [18,46]. As digital health literacy encompasses credibility appraisal, contextual application, decision-making, and communication beyond searching [47], the low “evaluation” and “self-expression” scores may reflect practical barriers to using digital healthcare. With increasing digital touchpoints (e.g., kiosks, apps, online appointments, and screening) [48], limited capacity to interpret information and communicate needs during digital interactions may widen disparities in healthcare access and outcomes for this population.

This pattern aligns with Bruland et al.’s review [23], which noted that digital health literacy research involving people with intellectual disabilities remains limited and has often focused on functional-level skills. In the present study, searching showed the highest mean score, whereas evaluation and self-expression showed relatively lower mean scores. However, differences among subdomain means were modest and were not statistically tested; therefore, these findings should be interpreted cautiously. The observed pattern suggests that transition-age young adults with developmental disabilities may experience relatively greater difficulty with appraising information credibility and communicating health-related needs than with basic information searching. Accordingly, interventions may consider going beyond basic search training to include supported practice in evaluating the credibility of online health information, identifying reliable websites, and communicating symptoms or needs through kiosks, hospital apps, and messaging platforms. Future studies should formally test subdomain differences and further examine the dimensionality of digital health literacy in this population.

These findings should also be interpreted in light of the mobile health context, as transition-age young adults may often access and communicate health information through smartphones, mobile applications, social media, and other mobile-based platforms [21]. Thus, mobile health may represent a key practical setting in which digital health literacy is applied. Future studies should more explicitly distinguish between general digital health literacy and mobile health literacy in this population. Another important interpretive issue is the distinction between independent and supported digital health literacy. Because only about half of the participants reported being able to search for health information independently, digital health literacy may not have functioned purely as an individual competency in this sample. For those who required assistance or could not search independently, information seeking, appraisal, communication, and decision-making may have been shaped by family members, teachers, facility staff, or other supporters. Reported digital health literacy should therefore be interpreted in light of both participants’ own competencies and the support environments in which their digital health activities took place. Interventions in this population may therefore need to target not only individual skills but also the supporters and environments that shape health-related digital engagement.

In this study, the mean perceived benefits score was 4.03 out of 5, suggesting a relatively high level of recognition of the benefits of maintaining a healthy lifestyle among transition-age young adults with developmental disabilities. Individuals with developmental disabilities often struggle with abstract concepts and anticipating behavioral consequences due to impairments in abstract thinking and judgment, as noted in the DSM-5-TR criteria for intellectual disability [38]. However, prior studies indicate that perceived benefits can remain high with appropriate support: adults with developmental disabilities show a strong desire for health management and positive perceptions of its benefits [42], and participatory approaches (e.g., photovoice) demonstrate their ability to articulate the advantages of healthy eating and activity in their own words [49]. These findings suggest that transition-age young adults with developmental disabilities can believe that “a healthy lifestyle benefits me,” potentially motivating health self-management. Consequently, the high perceived benefits observed in this study underscore the importance of supporting transition-age young adults with developmental disabilities not merely as passive service recipients but as active agents in managing their own health.

In this study, we controlled for gender, age, type of disability, and the ability to search for health information using digital devices—all identified in previous research as factors associated with health-promoting behaviors [15,22,39,40]—to estimate more accurately the associations of digital health literacy and perceived benefits with health-promoting behaviors. Physical and mental health conditions, however, were neither measured nor included as control variables. Individuals managing chronic or mental health conditions may be more motivated to seek health information and engage in preventive practices, so unmeasured differences in health status may have influenced the observed associations. These associations should therefore be interpreted with caution, given the possibility of confounding by health status [12,13,27].

Hierarchical regression showed that digital health literacy and perceived benefits accounted for additional variance in health-promoting behaviors beyond general characteristics, with perceived benefits showing the strongest association. Although previous empirical studies have also supported the relevance of perceived benefits to health-related behaviors or intentions, they were conducted among non-disabled children, college students, or older adults [50,51,52], and examined health behaviors, behavioral intention, or activity participation rather than health-promoting behaviors among people with developmental disabilities. Therefore, the present study extends previous evidence by showing that perceived benefits are strongly associated with self-reported health-promoting behaviors among transition-age young adults with developmental disabilities. Nevertheless, perceived benefits are best understood as only one motivational component, not as the sole determinant of health-promoting behaviors [27,28,53]. Future research should examine perceived benefits alongside health self-management capacity, environmental supports, and broader health-related beliefs, including perceived barriers, self-efficacy, perceived susceptibility, and perceived severity.

In Korea, research on digital health literacy among individuals with developmental disabilities has primarily focused on status and levels [22], with no studies examining its association with health-promoting behaviors. To our knowledge, this is among the first studies to show that digital health literacy among transition-age young adults with developmental disabilities is significantly associated with health-promoting behaviors, indicating that greater ability to access, understand, and use digital health information is associated with stronger health behaviors. Similar positive associations have been reported in university students, community-dwelling older adults, and general adult populations in Turkey [54,55,56] and among middle-aged women and nursing students in Korea [57,58]. Meta-analytic evidence indicates that eHealth literacy is positively associated with health behaviors and may contribute to behavior change [59]. Consistent with this, the present findings suggest that both digital health literacy and perceived benefits are relevant to health-promoting behaviors in this population. Because this study examined only direct associations in cross-sectional data, however, it cannot determine whether digital health literacy mediates or moderates the relationship between perceived benefits and health-promoting behaviors. Longitudinal studies should test more complex pathways, including whether digital health literacy helps translate health-related beliefs into health-promoting behaviors.

## 5. Conclusions

This study found that digital health literacy and perceived benefits were significantly associated with health-promoting behaviors among 137 transition-age young adults with developmental disabilities. After controlling for gender, age, type of disability, and independent digital health information-searching ability, both variables remained significantly associated with health-promoting behaviors, suggesting that digital competencies and motivational cognitions should be considered in health promotion and in digital and mobile health support for this population.

Several limitations should be noted. First, the cross-sectional design limits causal inference, and convenience sampling from service-registered institutions in one province limits the generalizability of the findings to all transition-age young adults with developmental disabilities in Korea. Second, we did not measure participants’ exposure to specific structured supports such as health education, independent living programs, transition preparation, or digital skills training. The observed associations may therefore reflect not only individual digital health literacy and perceived benefits but also differences in support provision. Third, we did not collect information on co-occurring physical and mental health conditions, or on detailed developmental or psychiatric diagnoses. These factors may influence health information seeking, motivation, engagement in health-promoting behaviors, and subgroup findings by disability type; their omission therefore limits the interpretation of the observed associations. Fourth, although the study used a developmental disability framework consistent with the Korean administrative and service context, most participants had intellectual disability. The findings may therefore apply more directly to transition-age young adults with intellectual disability than to the broader population of individuals with developmental disabilities, including those with autism spectrum disorder but no intellectual disability. Fifth, the use of comprehension-adapted measures may have limited the ability to capture broader health behaviors, such as nutrition, sleep, self-management, and service use. Sixth, self-report Likert-type measures may have introduced response bias, including acquiescence and social desirability, particularly for abstract or evaluative items among those with intellectual disabilities [60]. As all key variables were measured using self-report scales in the same survey, shared-method variance may have inflated the observed associations. Future studies should use multimethod or objective assessments [61]. Seventh, although recruitment setting type was included as a covariate, individual site identifiers were not retained in the analytic dataset. Therefore, we could not estimate cluster-robust standard errors or directly account for within-site non-independence among participants recruited from the same institution. This limitation may have led to underestimated standard errors, which should be considered when interpreting the statistical significance of the findings. Finally, because no qualitative data were collected and eligibility was limited to individuals who could complete the questionnaire independently, the study could not fully explain participants’ difficulties with higher-order digital health literacy skills or represent individuals with greater support needs.

Longitudinal or intervention studies with more diverse samples are needed to clarify causal pathways and to examine how digital health literacy, perceived benefits, health self-management capacity, environmental supports, broader health-related beliefs, and structured service experiences jointly shape health-promoting behaviors. Researchers should also develop and validate population-specific instruments and use qualitative or mixed-methods approaches to better capture the perspectives and support needs of this population.

## Figures and Tables

**Table 1:** Participants’ General Characteristics and Corresponding Health-Promoting Behaviors (*n* = 137).

Variable	Category	*n*	%	Health-Promoting Behaviors Mean ± SD	t/F	*p*
Gender	Male	86	62.8	3.85 ± 0.69	−1.36	0.177
	Female	51	37.2	4.01 ± 0.61		
Age (years)	19–20	45	32.8	4.00 ± 0.50	0.81	0.523
	21	31	22.6	3.86 ± 0.59		
	22	15	10.9	3.70 ± 0.86		
	23	13	9.5	4.05 ± 0.71		
	24	33	24.2	3.88 ± 0.78		
Type of disability	Intellectual disability	126	92.0	3.93 ± 0.65	0.83	0.410
	Autism spectrum disorder	11	8.0	3.76 ± 0.74		
Recruitment setting type	Special school	80	58.4	3.94 ± 0.53	0.49	0.622
	Social welfare facility	57	41.6	3.88 ± 0.81		
Per week digital devices use frequency	Never	6	4.4	3.78 ± 0.38	0.96	0.429
	1–2 times/week	17	12.4	4.09 ± 0.59		
	3–4 times/week	18	13.1	3.97 ± 0.69		
	Almost daily	65	47.5	3.95 ± 0.64		
	Several times a day	31	22.6	3.74 ± 0.74		
Frequency of searching for health information using digital devices	Never	54	39.4	3.84 ± 0.68	1.39	0.248
	About once a month	46	33.6	4.06 ± 0.55		
	2–3 times/month	26	19.0	3.78 ± 0.75		
	Once a week or almost daily	11	8.0	3.95 ± 0.68		
Ability to search for health information using digital devices	Able independently	68	49.6	4.04 ± 0.65	2.49	0.087
	Need assistance	37	27.0	3.78 ± 0.62		
	Unable independently	32	23.4	3.80 ± 0.69		

**Table 2:** Levels of Digital Health Literacy, Perceived Benefits, and Health-Promoting Behaviors (*n* = 137).

Variable	Mean ± SD (Item)	Range
Digital health literacy	2.47 ± 0.78	1–4
Searching	2.62 ± 0.86	1–4
Self-expression	2.38 ± 0.84	1–4
Evaluation	2.41 ± 0.84	1–4
Application	2.47 ± 0.85	1–4
Perceived benefits	4.03 ± 0.81	1–5
Health-promoting behaviors	3.91 ± 0.66	1–5
Personal hygiene and lifestyle habits	3.75 ± 0.75	1–5
Accident prevention	3.86 ± 0.79	1–5
Infectious disease prevention and management	4.15 ± 0.75	1–5
Mental health	3.98 ± 0.85	1–5

**Table 3:** Correlations among Digital Health Literacy, Perceived Benefits, and Health-Promoting Behaviors (*n* = 137).

Variables	Digital Health Literacy	Perceived Benefits	Health-Promoting Behaviors
	r (*p*)	r (*p*)	r (*p*)
Digital health literacy	1		
Perceived benefits	0.38 (<0.001) *	1	
Health-promoting behaviors	0.50 (<0.001) *	0.68 (<0.001) *	1

* *p* < 0.001.

**Table 4:** Factors Associated with Health-Promoting Behaviors (*n* = 137).

Variable	Model 1				Model 2			
	B	β	t	*p*	B	β	t	*p*
(Constant)	4.42		3.69	<0.001	2.71		3.22	0.002
Gender (female) †	0.13	0.10	1.07	0.286	0.07	0.05	0.79	0.434
Age (years)	−0.02	−0.05	−0.34	0.733	−0.07	−0.18	−1.80	0.075
Type of disability (autism spectrum disorder) †	−0.05	−0.02	−0.24	0.809	0.06	0.02	0.38	0.701
Recruitment setting type (social welfare facility) †	−0.01	−0.01	−0.06	0.949	0.17	0.13	1.32	0.188
Ability to search for health information using digital devices †								
Need assistance	−0.27	−0.18	−1.96	0.052	0.04	0.02	0.36	0.722
Unable independently	−0.20	−0.13	−1.42	0.157	0.23	0.15	1.91	0.058
Digital health literacy					0.31	0.37	4.65	<0.001
Perceived benefits					0.45	0.56	8.58	<0.001
R2 (ΔR2)	0.05 (0.05)				0.55 (0.50)			
Adj R2	0.01				0.52			
F (*p*)	1.14 (0.341)				19.70 (<0.001)			

† Male, intellectual disability, special school, and “able to search independently” were the reference categories for the dummy-coded variables.

## Data Availability

The data presented in this study are available on request from the corresponding author. The data are not publicly available because of privacy and ethical restrictions.

## Abbreviations

The following abbreviations are used in this manuscript:

**Abbreviation**	**Full form**
ANOVA	Analysis of Variance
COVID-19	Coronavirus Disease 2019
DHLI	Digital Health Literacy Instrument
DSM-5-TR	Diagnostic and Statistical Manual of Mental Disorders, Fifth Edition, Text Revision
I-CVI	Item-level Content Validity Index
IDD	Intellectual and Developmental Disabilities
S-CVI	Scale-level Content Validity Index
SD	Standard Deviation
